# Genetic Analysis Workshop 14: microsatellite and single-nucleotide polymorphism marker loci for genome-wide scans

**DOI:** 10.1186/1471-2156-6-S1-S1

**Published:** 2005-12-30

**Authors:** Joan E Bailey-Wilson, Laura Almasy, Mariza de Andrade, Julia Bailey, Heike Bickeböller, Heather J Cordell, E Warwick Daw, Lynn Goldin, Ellen L Goode, Courtney Gray-McGuire, Wayne Hening, Gail Jarvik, Brion S Maher, Nancy Mendell, Andrew D Paterson, John Rice, Glen Satten, Brian Suarez, Veronica Vieland, Marsha Wilcox, Heping Zhang, Andreas Ziegler, Jean W MacCluer

**Affiliations:** 1 Inherited Disease Research Branch, National Human Genome Research Institute, National Institutes of Health, Baltimore, Maryland 21224, USA; 2 Department of Genetics, Southwest Foundation for Biomedical Research, San Antonio, Texas, USA; 3 Mayo Clinic, Rochester, Minnesota, USA; 4 University of California Los Angeles, Los Angeles, California, USA; 5 Universitat Gottingen, Germany; 6 Department of Medical Genetics, University of Cambridge, UK; 7 University of Texas M.D. Anderson Cancer Center, Houston, Texas, USA; 8 National Cancer Institute, National Institutes of Health, Bethesda, Maryland, USA; 9 Mayo Clinic College of Medicine, Rochester, Minnesota, USA; 10 Case Western Reserve University, Cleveland, Ohio, USA; 11 Johns Hopkins School of Medicine, Baltimore, Maryland, USA; 12 University of Washington, Seattle, Washington, USA; 13 Center for Craniofacial and Dental Genetics, University of Pittsburgh, Pittsburgh, Pennsylvania, USA; 14 State University of New York at Stony Brook, Stony Brook, New York, USA; 15 The Hospital for Sick Children, Toronto, Canada; 16 Washington University School of Medicine, St. Louis, Missouri, USA; 17 Centers for Disease Control, Atlanta, Georgia, USA; 18 University of Iowa, Iowa City, Iowa, USA; 19 Boston University, Boston, Massachusetts, USA; 20 Yale University, New Haven, Connecticut, USA; 21 Universitat de Luebeck, Luebeck, Germany

## Preface

This supplement to *BMC Genetics *contains the proceedings of the Genetic Analysis Workshop 14 (GAW14), which was held September 7–10, 2004, in Noordwijkerhout, The Netherlands. These workshops have been held since 1982 and now are held biennially. They serve as a forum for statisticians, epidemiologists, geneticists, and other scientists interested in these fields to introduce novel statistical methods and to evaluate and compare novel and existing methods. At each GAW, an existing dataset is selected, and a set of simulated data is devised such that statistical questions of wide and current interest may be addressed. These data are made available to scientists worldwide who then report the results of their analyses of these data at the GAW meeting. GAW attendees must submit an analysis of one of these datasets, or be a workshop organizer or a dataset provider. The purpose of these workshops is to allow the comparison of statistical methodologies for genetic epidemiology using the same, well-described datasets. More information about GAW, including details of upcoming workshops, may be found at .

For GAW14, the overarching theme was comparison of microsatellite and single-nucleotide polymorphism (SNP) markers for genome-wide scans and the statistical methods that can best exploit the information provided in such scans for linkage and association studies. Two datasets were available for GAW14 participants to analyze. As is traditional at GAWs, one of these was a simulated dataset and one consisted of data from an actual human study. Attempts were made in the data simulation to mirror many of the characteristics of the real dataset, including a map of microsatellite markers plus a denser map of SNP markers that were available for fine-mapping. Both datasets are discussed briefly below, and more detailed descriptions can be found in Edenberg et al. [1] and Greenberg et al. [2].

The Collaborative Study on the Genetics of Alcoholism (COGA) generously donated extensive family data on alcoholism, related phenotypes, pertinent covariates and a set of previously analyzed genome-scan microsatellite marker genotypes for use in GAW14. COGA is a nine-site national collaborative study funded by the National Institute on Alcohol Abuse and Alcoholism (NIAAA) and the National Institute on Drug Abuse (NIDA) with the primary goal of identifying and characterizing genes that affect the susceptibility to develop alcohol dependence and related phenotypes. COGA has been committed to sharing data with the scientific community to expedite progress in understanding alcoholism and related phenotypes. COGA also provided data to GAW11, and has created an archival database of these families, with both phenotypic data and immortalized cell lines; these data are accessible to investigators for further study through NIAAA . Genome-wide linkage scans, using microsatellite markers, have been performed on both an initial dataset of 105 multigenerational pedigrees and a replication dataset with 157 multigenerational pedigrees. In a departure from GAW tradition, extensive new SNP genotyping was performed on DNA provided by COGA for the previously genotyped families. Illumina, Affymetrix, and the Center for Inherited Disease Research (CIDR) performed this work and donated these data to GAW14 and to COGA. A subset of 143 informative families, selected from the initial and replication datasets, were genotyped for these SNP markers. A total of 1,350 COGA samples were genotyped by Affymetrix for 11,560 SNPs from their GeneChip Mapping 10 K array and by Illumina for their Linkage III Panel containing 4,763 SNP markers. These data are described in detail in Edenberg et al. [1].

The simulated data were designed to have similarities to the real dataset. It was assumed that a complex trait such as alcoholism might have both genetic and environmental risk factors. It was further assumed that such a complex trait might be defined/measured in a variety of different ways by different investigators, have associated endophenotypes that are common in the general population, and is likely to be not one disease but a heterogeneous collection of clinically similar, but genetically distinct, entities. Disease characteristics and parameters were constant throughout all the simulations, but four different "studies" were simulated used varying ascertainment schemes based on differing assumptions about disease characteristics. One of the studies contained multiplex two and three generation pedigrees with at least four affected members. The simulated disease was a psychiatric condition with many associated behaviors (endophenotypes), almost all of which were genetic in origin. The underlying disease model contained four major genes and two modifier genes. The four major genes interacted with each other to produce three different "phenotypes", which were themselves heterogeneous. The population parameters were calibrated so that the major genes could be discovered by linkage analysis in most datasets. The association evidence was more difficult to calibrate but was designed to find statistically significant association in 50% of datasets. Some linkage disequilibrium between marker loci was simulated around some of the genes and also in areas without disease genes. Data distributed to participants contained about 1,000 SNPs and 400 microsatellite markers. Data obtainable via the internet consisted of a finer 10,000 SNP map, which also contained data on controls. These data are described in detail in Greenberg et al. [2].

In the spring of 2004, the availability of the GAW14 data was announced by e-mail to the more than 2,000 individuals on the GAW mailing list. A total of 129 groups requested GAW14 data. The COGA data were requested by 95 groups and the simulated data by 88 groups; 88 groups requested both datasets. In the summer of 2004, 183 contributed papers were received describing analyses of these datasets. A book and CD containing these contributions plus papers describing the datasets were distributed to workshop participants.

A total of 232 individuals from 14 countries attended GAW14. Attendees included investigators from four continents: Asia, Australia, Europe, and North America. The 183 contributions submitted to GAW14 were organized into 18 presentation groups of 7 to 15 papers each. The papers were grouped based on common methodological themes. Because the datasets were similar in many important aspects, and could be used to explore similar analytical problems, most presentation groups were assigned with no regard to the dataset analyzed. Thus, many of the groups of papers include analyses of both the simulated data and the COGA data. Within each presentation group, a co-author with previous GAW experience was asked to serve as group leader to facilitate group discussion, organize an oral presentation for the group, and take the lead in writing the group summary papers that will be published in *Genetic Epidemiology*. The 18 presentation groups were organized around common methodological issues: comparisons of SNPs versus microsatellite markers in linkage studies, integration of SNPs and microsatellites in linkage and association studies, linkage mapping methods, quantitative trait mapping, fine mapping, methods for generating haplotypes and identifying haplotype-tagging SNPs, approaches to dealing with linkage disequilibrium, methods for association mapping, methods for case-control analysis and multivariate analyses, applications of these methods to analysis of alcoholism, smoking and related traits in the COGA data, methods for data mining, methods for dealing with genetic heterogeneity, methods for detecting gene × gene interaction, approaches to dealing with genotyping errors, pedigree errors and missing data, and methods to test for parent of origin, genomic imprinting, mitochondrial and X-linked effects in genome scans. Each presentation group met individually during the workshop and members of most groups communicated beforehand to begin comparing and contrasting the approaches taken and the results obtained by group members. At GAW14, many groups used part of their group meeting time to allow individual investigators to present their work. These group meetings were mostly attended by group participants but were open to all GAW14 attendees. From this process, each group developed an oral presentation, summarizing and synthesizing the work of the individual papers, which was delivered to the full workshop audience during the general sessions. Individual contributions were also presented in the form of 59 posters displayed during four poster sessions.

The 163 papers included in this supplement to *BMC Genetics *are a subset of the 183 contributions presented at GAW14. All of these papers have been reviewed for scientific merit. The proceedings begin with two papers describing the two datasets, followed by the 163 individual GAW14 contributions organized by presentation group and alphabetically by first author within each group. In addition to the individual papers in this volume, each presentation group has a summary in a forthcoming supplement to the journal *Genetic Epidemiology *in which the present manuscripts are compared and contrasted and the important themes and results from each group are described. Novel methods for linkage and association analyses using SNPs and microsatellites were developed, compared to existing methods, and applied to the GAW data, resulting in many interesting conclusions concerning appropriate approaches to the analysis of these sorts of data and also concerning areas of methodological development that are currently needed in this field.
